# Association between stroke and fracture and the mediating role of depression: a cross-sectional study from NHANES 2017 to 2020

**DOI:** 10.3389/fneur.2025.1533565

**Published:** 2025-02-05

**Authors:** Yuqin Dan, Xuewen Pei, Danghan Xu, Zhaoxi Liu, Yuqi Wang, Meng Yin, Li Li, Gongchang Yu

**Affiliations:** ^1^Shandong University of Traditional Chinese Medicine, Rehabilitation Medicine School, Jinan, China; ^2^State University of New York at Buffalo, Albany, NY, United States; ^3^The First Affiliated Hospital of Guangzhou University of Chinese Medicine, Rehabilitation Center, Guangzhou, China; ^4^Affiliated Traditional Chinese Medicine Hospital of Guangzhou Medical University, Guangzhou, China; ^5^Neck-Shoulder and Lumbocrural Pain Hospital of Shandong First Medical University, Shandong First Medical University & Shandong Academy of Medical Sciences, Jinan, China; ^6^The Second Affiliated Hospital of Shandong University of Traditional Chinese Medicine, Rehabilitation Medicine Department, Jinan, China; ^7^The Central Laboratory, Shandong Mental Health Center, Shandong University, Jinan, China

**Keywords:** stroke, fracture, depression, association, NHANES, mediation

## Abstract

**Background:**

Stroke is a significant health threat, and its complex interplay with fractures warrants further investigation. Depression, a critical psychological mediator in various health conditions, may also play a role. This study aims to clarify the intricate relationships among stroke, depressive symptoms, and fracture risk, potentially informing more holistic clinical strategies.

**Methods:**

Utilizing the most recent data from the National Health and Nutrition Examination Survey (NHANES, 2017 to 2020), this study encompassed 4,979 valid samples. *T*-test and chi square test are conducted to compare the differences between fracture and non fracture subgroups. Subsequently, regression models were applied to assess the mediating impact of depression, with Sobel’s test and the bootstrap method deployed to substantiate the mediation pathways.

**Results:**

In this study, we conducted subgroup and regression analyses to investigate factors influencing fractures in stroke patients using NHANES data. Subgroup analysis revealed significant associations with gender, race, osteoporosis, and depression. Female stroke patients had a higher fracture rate (73.86% vs. 47.78%, *p* < 0.001), and those with post-stroke depression (29.67% vs. 13.16%, *p* < 0.001) or osteoporosis (33.33% vs. 15.81%, *p* < 0.05) were at increased risk of fractures. Logistic regression models showed a positive association between stroke and fractures in the unadjusted (OR = 1.862, 95% CI: 1.348–2.573, *p* < 0.001) and adjusted I models (OR = 1.789, 95% CI: 1.240–2.581, *p* < 0.01), but not in the adjusted II model. Depression was significantly correlated with fractures in all models (unadjusted OR = 2.785, 95% CI: 1.271–6.101, *p* < 0.05; Model 1 OR = 3.737, 95% CI: 1.470–9.498, *p* < 0.01; Model 2 OR = 3.068, 95% CI: 1.026–9.175, *p* < 0.05). Mediation analysis using Sobel and bootstrap tests indicated that depression mediates 7.657% of the relationship between stroke and fractures (*Z* = 2.31, *p* < 0.05), with significant indirect (*Z* = 2.80, *p* < 0.01), direct (*Z* = 3.61, *p* < 0.001), and total effects (*Z* = 3.92, *p* < 0.01). The direct effect of stroke on fracture was 0.079 (95% CI: 0.036–0.121), the total effect was 0.085 (95% CI: 0.043–0.128), and the indirect effect mediated by depressive symptoms was 0.007 (95% CI: 0.002–0.011). These results suggest that depressive symptoms following stroke may contribute to an increased risk of fractures.

**Conclusion:**

Depressive symptoms serve as a critical mediator in the link between stroke and fracture risk. Consequently, our study concludes that holistic prevention strategies for fractures in stroke patients must incorporate a focus on mental health to effectively address this complex clinical challenge.

## 1 Introduction

Stroke is now the second leading cause of death and disability worldwide (1). Data indicates a concerning trend of stroke incidence shifting toward younger demographics (2). Moreover, a staggering 70% of stroke-related fatalities and 87% of resulting disabilities are concentrated in low- and middle-income nations (3). This alarming situation not only leads to a significant loss of labor force but also imposes a substantial financial and healthcare burden on these countries (4). Fracture represents a perilous complication arising from stroke (5). The occurrence of fractures in the stroke population can hinder functional recovery by leading to prolonged hospitalization, reduced independence and delays in rehabilitation (6). Therefore, prevention of fractures is important for the recovery of stroke patients.

Depression affects approximately 71% of stroke survivors within the critical three-month post-stroke period (7). This mental health condition is a significant contributor to the exacerbation of physical decline and the decline in cognitive-psychological performance among individuals who have experienced a stroke (8–10). Notably, a negative correlation exists between the depression severity and bone density, with severe depression contributing to osteoporosis, and increased fracture risk (11, 12). The mechanisms underlying these phenomena are likely closely related to the regulation of the “brain-neuro-musculoskeletal” axis, which involves the bidirectional interaction between the brain’s neural control over the musculoskeletal system and the feedback regulation of brain function by the musculoskeletal system (13). For instance, the hypothalamic–pituitary–adrenal (HPA) axis, a critical pathway through which the nervous and endocrine systems interact, leads to the secretion of cortisol and other hormones upon activation (14). Depression often activates the HPA axis, increasing cortisol levels. Elevated cortisol not only exacerbates mood disorders like depression but also impairs musculoskeletal health by inhibiting osteoblast activity, reducing calcium absorption, and weakening bone structure. Cortisol plays a key role in stress responses, including fear, anxiety, and depression, while also inhibiting osteoblast activity, bone calcium absorption, and vascularization (15, 16). These effects indirectly disrupt bone microstructure and impedes bone turnover and metabolism (17). Therefore, depression, as a prevalent mood disorder, may significantly influence the link between stroke and fracture.

However, current research on the association between neuropsychiatric disorders and musculoskeletal diseases primarily focuses on adolescent and elderly populations (18–20). The relationship between depression and fracture incidence in stroke patients has not been well-defined. While previous studies have reported significant associations between depression and fall risk in stroke patients, most have been limited by small sample sizes and insufficient analytical depth, leaving the precise role of depression in the relationship between stroke and fracture poorly understood (21). To address these limitations, this study employs robust statistical methods, such as Sobel’s test and bootstrap analysis, which enhance the reliability and validity of the mediation findings. This provides a solid foundation for future research and clinical practice, supporting the development of more effective strategies (22, 23).

In summary, to delve deeper into the association between stroke and fracture incidence risk, we used data from the National Health and Nutrition Examination Survey (NHANES) 2017–2020 to (1) identify the influencing factors affecting fracture incidence in stroke and non-stroke populations; (2) investigate the relationship between stroke and fracture incidence and analyze the mediating role and extent of the influence of depression between stroke and fracture; (3) analyze stroke and fracture incidence-related Potential Mechanisms.

## 2 Materials and methods

### 2.1 Study population

A cross-sectional study was conducted using data from the National Health and Nutrition Examination Survey (NHANES) official website: https://www.cdc.gov/nchs/nhanes/NHANES. First, we included the entire 2017–2020 NHANES population (*n* = 15,560). Samples younger than 50 (*n* = 104,573) and those with missing fracture information (*n* = 8) were then excluded. We finally analyzed the remaining sample of 4,979 cases. Due to the fact that Mexican American persons were oversampled and all Hispanic persons from 2007 onwards were oversampled, we adjusted all analyses for the complex sample design of NHANES using the sample weights from NHANES (24).

### 2.2 Outcome variable

The primary outcome variable is fracture. Participants were asked, “Has the doctor ever told you about a hip/wrist/spine fracture or fracture?” A fracture in any of these three areas is considered a separate issue; a ‘yes’ to any one of them is indicative of a fracture (25).

### 2.3 Evaluation of exposures

The exposure variable is stroke, which was diagnosed through self reporting by doctors and patients in face-to-face interviews. A person who answers “Yes” to the question “Ever told you had a stroke?” is defined as having a stroke. With the NHANES database’s limited details on stroke types and severity, it is reasonable to deduce that most stroke participants in this study likely suffer from ischemic stroke, aligning with the prevailing research within the NHANES database (26).

Depression is the main covariate. The Patient Health Questionnaire (PHQ-9) is used to diagnose depression, which is a self reporting assessment based on the fourth edition of the Diagnostic and Statistical Manual of Mental Disorders (DSM-IV) version, used to describe the nine signs and symptoms of depression. The scores were summed to give each participant a total score ranging from 0 to 27. As in previous studies, the present study defined depression as a total PHQ-9 score of ≥10 (27).

In addition, covariates also include: osteoporosis, dizziness, fasting glucose, alcohol, diabetes, High blood pressure, BMI, age, gender, race, marital status, education level, family monthly poverty level category, et al. An individual who affirms the query “Have you ever been diagnosed with osteoporosis or brittle bones?” is classified as having osteoporosis. Similarly, an individual who responds affirmatively to the question “Have you experienced dizziness or lightheadedness?” is categorized as experiencing dizziness. Furthermore, an individual who answers “Yes” to the question “Have you ever consumed any form of alcohol?” is identified as an alcohol consumer. Additionally, serum cotinine levels are a reliable biomarker for assessing tobacco exposure. Cotinine, a primary metabolite of nicotine, serves as an effective indicator of an individual’s level of exposure to tobacco smoke (28). All variables were obtained through standardized clinical evaluation and laboratory testing, and were selected based on previous literature and clinical expert opinions to control for potential confounding factors.

### 2.4 Ethic statement

The study protocol was reviewed and approved by the Research Ethics Review Board of the National Center for Health Statistics.

### 2.5 Statistical analysis

**Figure 1:**
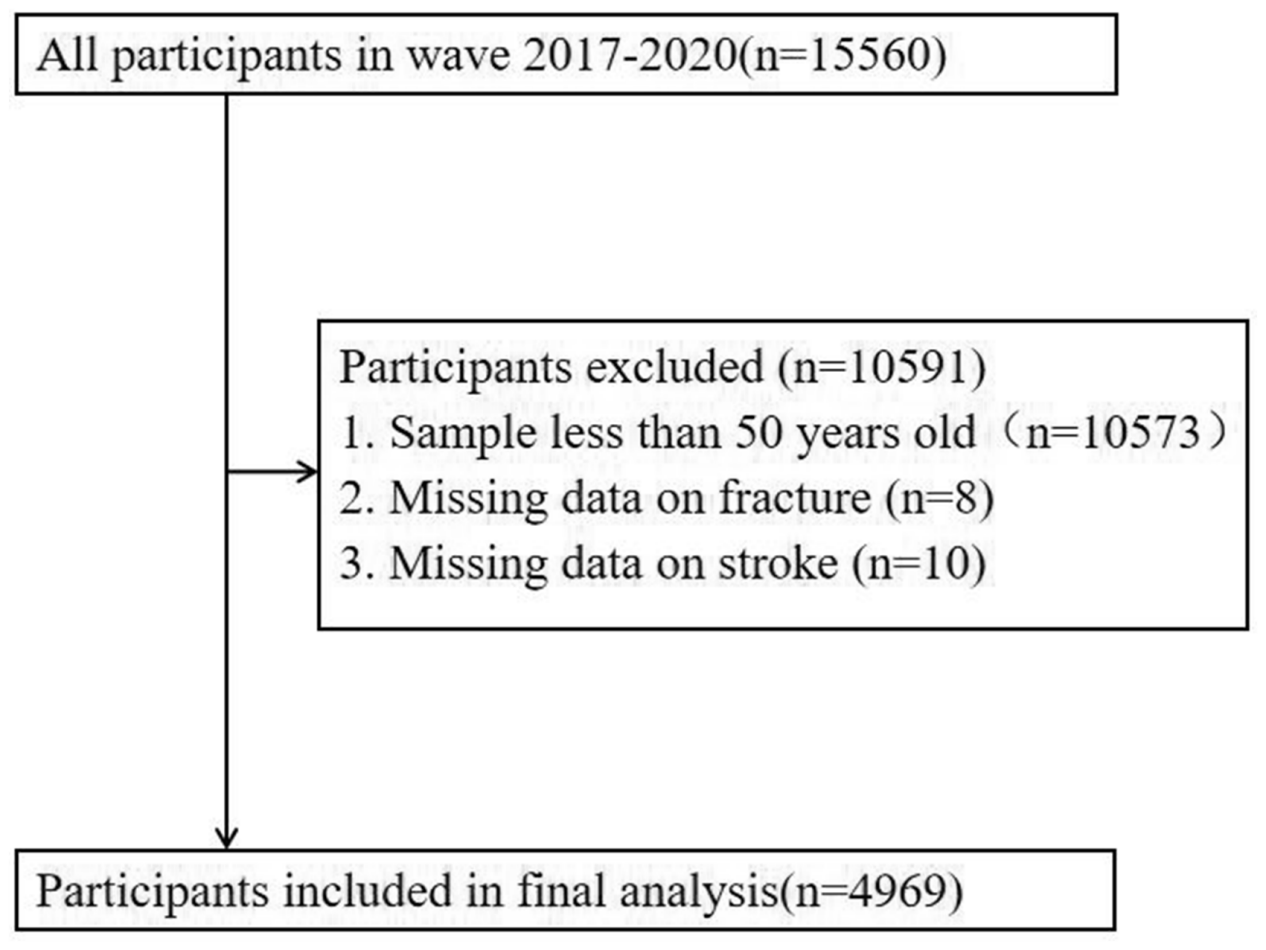
Flow chart.

We conducted all statistical analyses using SAS version 9.4. Firstly, this study conducted a statistical description of the demographic characteristics and key variables of society. Categorical variables are described by frequency (percentage), while continuous variables are described by mean (standard deviation, SD). The differences between fractured and non fractured subgroups were compared using *t*-test or chi square test. Secondly, a univariate analysis was conducted on the fracture and non fracture subgroups of stroke patients using the aforementioned method. All samples were weighted to account for potential biases and to ensure that the study’s findings are representative of the broader population. This approach is crucial for enhancing the accuracy and generalizability of the research results (29). Then, we employed weighted logistic regression models to estimate the adjusted odds ratios (aOR) for fracture occurrence, accounting for various covariates and adjusting for independent variables across different demographic groups. Model 1 focused on the influence of age, gender, and race, while Model 2 expanded the analysis to include education, marital status, family monthly poverty level category, cotinine levels, and history of alcohol consumption. These additional variables in Model 2 aimed to provide a more comprehensive understanding of the factors associated with fracture risk. Both models were designed to control for potential confounding effects, ensuring that the estimated odds ratios were adjusted for the influence of these independent variables (30). Next, Judd and Kenny’s recommendations and Baron and Kenny’s causal step approach were employed to investigate the relationship between stroke, depression, and fracture among the population aged 50 and above.Therefore, the three regression processes would estimate the follow ing effects: (1) the effect of stroke on fracture; (2) the effect of stroke on depressive symptoms; (3) the effect of depressive symptoms on fracture when stroke were controlled; and (4) the effect of stroke on fracture when depressive symptoms were controlled (31). In our analyses, covariates were adjusted to enhance model estimation precision. The Sobel test was applied to assess the significance of the indirect effect, with a Z-value surpassing 1.96 indicating significance. Effect sizes and 95% confidence intervals were determined using bootstrapping with 5,000 resamples, considering effects significant if their intervals did not span zero. Statistical tests were two-tailed, with *p* < 0.05 set as the threshold for significance (25) (Figure 1).

## 3 Result

### 3.1 Univariate analysis

The results of univariate analysis are shown in Table 1. We found significant association between participants with and without fractures in terms of age, race, marital status, education level, tobacco smoke exposure, alcohol consumption, bone density level, depression status, and stroke. Compared with the non fracture group, the fracture group had a higher proportion of patients diagnosed with stroke (10.93% > 6.18%, *p* < 0.001), depression (11.13% > 6.21%, *p* < 0.05), and osteoporosis (21.63% > 11.51%, *p* < 0.001). However, there is no significant association between dizziness and fractures(44.35% > 39.91%, *p* > 0.05).

**Table 1:** Weighted univariate analysis of factors affecting fractures.

Variable	Fracture		*p*-value
	Yes	No	
No. of participants	726	4,253	
Age, years	65.32 (0.58)	63.87 (0.29)	**0.0085**
Gender, %			0.7979
Male	366 (46.91)	2094 (46.19)	
Female	360 (53.09)	2,159 (53.81)	
Race, %			**<0.0001**
Mexican American	38 (2.30)	419 (5.37)	
Other Hispanic	73 (4.92)	432 (6.45)	
Non-Hispanic White	403 (80.71)	1,558 (67.33)	
Non-Hispanic Black	134 (5.42)	1,198 (11.40)	
Non-Hispanic Asian	35 (1.97)	494 (5.83)	
Other Race - Including Multi-Racial	43 (4.66)	152 (3.62)	
Marital status, %			**0.0053**
Married/Living with Partner	376 (58.64)	2,444 (64.94)	
Widowed/Divorced/Separated	291 (36.24)	1,435 (28.76)	
Never married	59 (5.12)	366 (6.30)	
Education level			**0.0441**
Less than 9th grade	55 (2.69)	448 (5.24)	
9-11th grade (Includes 12th grade with no diploma)	74 (5.95)	508 (7.77)	
High school graduate/GED or equivalent	199 (31.01)	1,060 (28.73)	
Some college or AA degree	249 (34.55)	1,234 (28.17)	
College graduate or above	148 (25.7880)	992 (30.08)	
Family monthly poverty level category			0.8509
Monthly poverty level index =1.30	177 (17.6039)	1,086 (18.85)	
1.30 < Monthly poverty level index = 1.85	100 (12.4898)	597 (12.61)	
Monthly poverty level index >1.85	363 (69.9063)	2009 (68.54)	
BMI (kg/m**2)	30.02 (0.4)	29.87 (0.16)	0.7253
High blood pressure	446 (54.09)	2,345 (49.41)	0.1472
Fasting Glucose (mg/dL)	114.926560 (2.09)	117.135401 (1.55)	0.3133
Diabetes	183 (20.27)	1,019 (19.19)	0.6496
Cotinine, Serum (ng/mL)	53.651996 (4.43)	40.818935 (2.63)	**0.0068**
Alcohol	592 (95.75)	3,263 (91.28)	**0.0028**
Osteoporosis	160 (21.63)	450 (11.51)	**<0.0001**
Depression	88 (11.13)	317 (6.21)	**0.0013**
Dizziness	133 (44.35)	567 (39.91)	0.2357
Stroke	101 (10.93)	334 (6.18)	**<0.0001**

Bolded text reads: *p* < 0.05.

### 3.2 Subgroup analysis

Subgroup analysis was conducted to confirm which factors affect fractures in the stroke population. The results of subgroup analysis are shown in Table 2. We found a significant association between gender, race, osteoporosis, depression, and fractures in stroke patients. Specifically, among stroke patients, the proportion of male fractures is lower (26.14% < 52.22%, *p* < 0.001), while the probability of female fractures is higher (73.86% > 47.78%, *p* < 0.001). In addition, stroke patients with depression(29.67% > 13.16%, *p* < 0.001) and osteoporosis(33.33% > 15.81%, *p* < 0.05) are more likely to experience fractures.

**Table 2:** Weighted univariate analysis of the impact on fractures in stroke patients.

Variable	Stroke		
	Fracture	Non-fracture	*p*
No. of participants	101	334	
Age, years	68.65 (1.53)	68.01 (0.83)	0.734
Gender, %			
Male	47 (26.14)	180 (52.22)	**<0.0001**
Female	54 (73.86)	154 (47.78)	
Race, %			
Mexican American	4 (1.65)	19 (4.33)	**0.0122**
Other Hispanic	8 (2.59)	24 (5.68)	
Non-Hispanic White	54 (77.69)	131 (64.51)	
Non-Hispanic Black	21 (7.94)	130 (18.16)	
Non-Hispanic Asian	4 (1.64)	15 (2.75)	
Other Race	10 (8.48)	15 (4.57)	
Marital status, %			
Married/Living with Partner	47 (55.34)	158 (56.42)	0.4007
Widowed/Divorced/Separated	44 (35.17)	148 (39.26)	
Never married	10 (9.49)	26 (4.32)	
Education level			
Less than 9th grade	10 (4.76)	30 (6.63)	0.2507
9-11th grade (Includes 12th grade with no diploma)	11 (6.51)	61 (13.46)	
High school graduate/GED or equivalent	35 (37.87)	107 (40.62)	
Some college or AA degree	27 (24.61)	88 (24.43)	
College graduate or above	18 (26.25)	48 (14.86)	
Family monthly poverty level category			
Monthly poverty level index =1.30	34 (31.55)	111 (25.51)	0.7839
1.30 < Monthly poverty level index = 1.85	15 (13.30)	45 (13.92)	
Monthly poverty level index >1.85	40 (55.15)	138 (60.57)	
BMI (kg/m**2)	30.72 (0.72)	29.85 (0.55)	0.316
High blood pressure			
Yes	76 (71.83)	261 (71.64)	0.9881
No	25 (28.17)	73 (28.36)	
Fasting Glucose (mg/dL)	120.71 (8.55)	127.59 (6.56)	0.5116
Diabetes			
Yes	43 (43.64)	106 (32.23)	0.3055
No	55 (54.43)	216 (66.46)	
Borderline	3 (1.92)	11 (1.31)	
Cotinine, Serum (ng/mL)	52.37 (13.87)	65.19 (13.71)	0.5708
Alcohol			
Yes	78 (87.44)	253 (93.2069)	0.2654
No	8 (12.55)	25 (6.7931)	
Osteoporosis			
Yes	34 (33.33)	46 (15.81)	**0.0167**
No	67 (66.67)	286 (84.19)	
Dizziness			
Yes	34 (56.58)	88 (54.98)	0.8836
No	15 (43.42)	75 (45.02)	
Depression			
Yes	25 (29.67)	48 (13.16)	**0.0028**
No	76 (70.33)	286 (86.84)	

Bolded text reads: *p* < 0.05.

### 3.3 Logistic regressive analysis

The associations between stroke, depression, and fracture risk were evaluated using Unadjusted, Model I, and Model II approaches. The effect sizes, ORs, and 95% CIs are presented in Table 3. The results showed that stroke was positively associated with fracture risk in both Unadjusted model (OR = 1.862, 95% CI: 1.348–2.573, *p* < 0.001) and Model I (OR = 1.789, 95% CI: 1.240–2.581, *p* < 0.01). However, there is no significant association in Model II. In the stroke population, using non depressed individuals as a reference, there was a significant positive association between depression and fractures among Unadjusted (OR = 2.785, 95% CI: 1.271–6.101, *p* < 0.05), Model I (OR = 3.737, 95% CI:1.470–9.498, *p* < 0.01) and Model II (OR = 3.068, 95% CI: 1.026–9.175, *p* < 0.05).

**Table 3:** Multi-model regression analysis of fracture risk in stroke patients by depression status.

Variable	Unadjusted		Model I		Model II	
	aOR (95%CI)	*p*	aOR (95%CI)	*p*	aOR (95%CI)	*p*
Stroke						
Yes	1.862 (1.348,2.573)	**0.0005**	1.789 (1.240,2.581)	**0.0031**	1.624 (0.931,2.832)	0.0845
No	Referent		Referent		Referent	
Stroke						
With depression	2.785 (1.271,6.101)	**0.0125**	3.737 (1.470,9.498)	**0.0075**	3.068 (1.026,9.175)	**0.0453**
Without depression	Referent		Referent		Referent	

Bolded text reads: *p* < 0.05.

### 3.4 Mediation analysis

**Figure 2:**
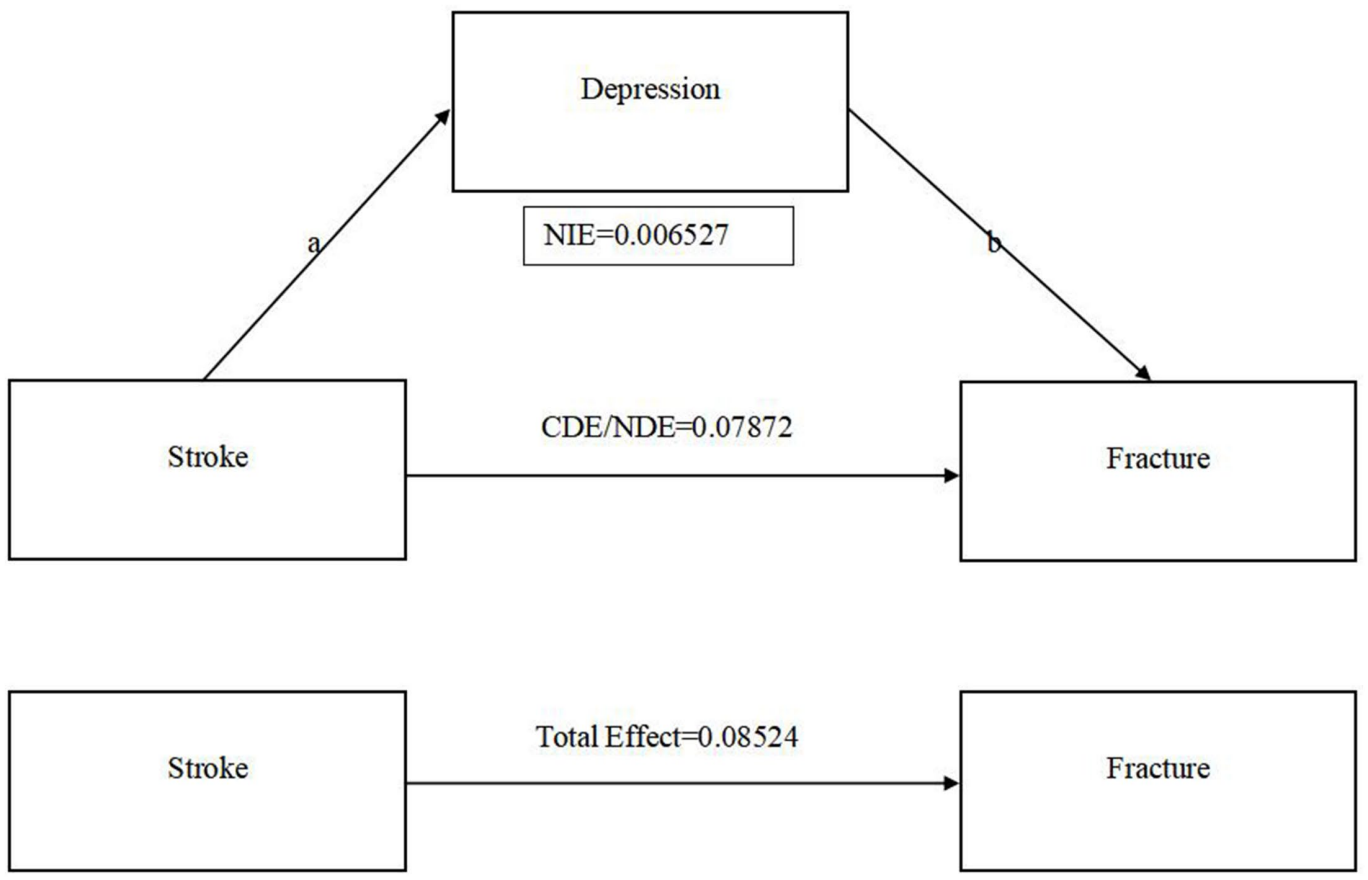
The relationship between stroke, depression and fracture. (a) CDE: Controlled Direct Effect; NDE: Natural Direct Effect; NIE(a*b): Natural Indirect Effect. (b) Adjusted covariates osteoporosis/age/gender/race/marital status/education level/family monthly poverty level category/tobacco smoke exposure/alcohol.

Regression analysis was utilized to explore the mediating role of depressive symptoms on the relationship between stroke and fracture. Meanwhile, we adjusted the covariate osteoporosis, age, gender, race, marital status, education level, family monthly poverty level category, tobacco exposure and alcohol. The Sobel and bootstrap tests were conducted to check for indirect, direct, and total effects. Table 4 shows that the percentage of depression mediated stroke and fracture is 7.657% (*Z* = 2.31, *p* < 0.05). As shown in Figure 2, Sobel test indicates that indirect effects (*Z* = 2.80, *p* < 0.01), direct effects (*Z* = 3.61, *p* < 0.001), and total effective rate (*Z* = 3.92, *p* < 0.001) are significant. This indicates that stroke can cause depressive symptoms, thereby increasing the risk of fractures. The boot strap method indicated that the direct effect of stroke on fracture was 0.079 (95% CI: 0.036–0.121), while the total effect was 0.085 (95% CI: 0.043–0.128). The indirect effect of stroke on fracture mediated by depressive symptoms was 0.007 (95% CI: 0.002–0.011).

**Table 4:** Mediation analysis of depression on stroke and fracture.

	Estimate	Std. Error	95% Confidence interval		Z	*p*
			Lower	Upper		
Stroke→depression→fracture	7.657	3.314	2.21	11.83	2.31	**0.0209**

Adjusted covariates osteoporosis/age/gender/race/marital status/education level/family monthly poverty level category/tobacco smoke exposure/alcohol. Bolded text reads: *p* < 0.05.

## 4 Discussion

Using data from 2017 to 2020 in the National Health and Nutrition Examination Survey (NHANES), the study found a significant association between stroke and fractures, with depressive symptoms mediating the risk of fractures in stroke patients (32). Sobel and Bootstrap methods supported the results of fundamental analysis, demonstrating the robustness of the results (33).

The relationship between stroke and fracture is influenced by multiple factors. While stroke survivors are prone to dizziness, often leading to falls and fractures (34, 35). However, the present study found no significant association between dizziness and fracture risk in this population. This suggests that dizziness may not be a primary factor in stroke-related fractures (36). As is well known, osteoporosis is the primary factor leading to fractures (37). The study identified a significant association between osteoporosis and fractures in stroke patients, likely due to limited mobility, reduced mechanical load from paralysis (38), and oxidative stress from cerebral ischemia–reperfusion injury. This stress disrupts the dynamic balance of osteoblast differentiation, apoptosis, and metabolism, contributing to osteoporosis (39). At the same time, gender emerges as an important influencing factor (40). Table 2 shows that in the stroke population, women in the stroke population are more prone to fractures than men. Yao et al. (41) reveal that estrogen fluctuations during menopause disrupt neurotransmitter secretion, affecting osteoblast and osteoclast balance and leading to osteoporosis. Furthermore, estrogen may contribute to osteoporosis progression by inducing oxidative stress, triggering inflammatory responses, and modulating microRNA expression, further increasing fracture susceptibility (42).

Existing research confirms that depression plays a pivotal mediating role in stroke and its associated complications (43), with a significant association between stroke and depression (44). In addition, osteoporosis can be triggered by increased life stress and depression (45). Hence, we cautiously posit that depression could serve as a critical intermediary in the relationship between stroke and fractures. Table 3 shows that the stroke and unadjusted fracture models showed significant associations. Nonetheless, following the partial adjustment for confounding factors, the association observed between stroke and fracture models became non-significant. This indicates that the association between stroke and fractures is likely due to a complex interplay of various contributing elements (46). However, stratified regression analyses of the stroke population yielded a significant association between depressive status and the fracture model in the stroke population, whether or not confounders were excluded (47). Therefore, depression plays an important role in the relationship between stroke and fracture. Table 4 indicates that while the mediated association between depression and the occurrence of stroke-related fractures is modest (7.57%, *p* < 0.05) but statistically significant (48). The Sobel test results, as illustrated in Figure 2, confirm the statistical significance of the indirect effects (*Z* = 2.80, *p* < 0.01), direct effects (*Z* = 3.61, *p* < 0.001), and total effects (*Z* = 3.92, *p* < 0.001) of stroke on depressive symptoms and subsequent fracture risk. Bootstrap analysis provides a more nuanced view, pinpointing the direct effect of stroke on fractures at 0.079 (95% CI: 0.036–0.121) and the total effect at 0.085 (95% CI: 0.043–0.128). The indirect effect, mediated by depression, is a modest but significant 0.007 (95% CI: 0.002–0.011). These insights highlight the pivotal role of depression as a mediator in the relationship between stroke and fracture risk, underscoring the need for integrated approaches in managing these conditions (49).

In addition to exploring depression as a mediator between stroke and fracture risk, the underlying neurophysiological mechanisms warrant further investigation. Stroke induces structural and functional impairments in critical brain regions, which can precipitate negative emotional states such as depression (50). This emotional disturbance is associated with dysregulation of neurotransmitter secretions (51). Specifically, the HPA axis increases cortisol levels while decreasing serotonin levels (52). These imbalances impair osteoblast function, hinder calcium absorption in bones, and impede skeletal angiogenesis (53). Consequently, bone microarchitecture is compromised, and normal bone turnover and metabolism are disrupted, predisposing individuals to osteoporosis and an increased risk of fractures. Conversely, fractures also impact stroke recovery (47). Research indicates that fractures can enhance inflammation in the peri-infarct area, thereby exacerbating ischemic stroke (54). Furthermore, inadequate post-fracture care leading to lower extremity deep vein thrombosis also increases the risk of stroke (55). Thus, stroke and fractures may have a reciprocal causal relationship, with regulatory mechanisms based on the “brain-neuro-musculoskeletal” axis.

During the analysis, we searched for papers published on PubMed before September 2024 using the keywords “stroke,” “fracture,” and “depression.” A total of 199 papers were retrieved, of which three were relevant to our study. Yeh et al. (56) found that post-stroke depression significantly contributes to hip fractures among stroke survivors, exerting a disproportionately negative effect on younger individuals, irrespective of gender and the presence of comorbid conditions. Kelly et al. (57) consider that the brain-bone axis plays a crucial role in the regulation of skeletal metabolism, sensory innervation, and endocrine crosstalk among these organs. Furthermore, a previous study identified a notable association between stroke and fractures in elderly patients undergoing rehabilitation post-stroke (58). However, unlike our findings, no significant disparities in depressive symptoms and functional status were observed upon admission and discharge among the compared patient cohorts. This discrepancy may be related to the failure to exclude confounding factors (59). Additionally, the previous study did not quantify depression’s influence on the stroke-fracture relationship. Moreover, it lacked an analysis of the underlying mechanisms at play.

Our research has to some extent overcome these shortcomings and has the following advantages. Firstly, we established a distinct association between stroke and fractures, particularly noting a significant association between fracture incidence and depression in stroke patients. Secondly, we employed a weighted regression model to scrutinize the relationship between depression and fractures in this patient population, effectively addressing the challenge of a limited sample size. Thirdly, through an in-depth analysis with depression as a mediating variable, we discovered that while depression’s contribution is not substantial, its statistical significance is marked. Lastly, we propose an interactive relationship exists between brain neurotransmitters and bone cells, potentially regulated by the ‘brain-bone’ axis.

However, our study has some limitations. The sample size is relatively small and the data is restricted to the American demographic, primarily composed of Hispanic Americans, the elderly, and children, which may affect the generalizability of the findings. Additionally, the stroke situation is obtained through self-report, which could easily cause recall bias. Moreover, NHANES only records fracture data for individuals over 50 years old, preventing us from observing stroke and fracture incidence in younger populations.

The cross-sectional design of our study limits the ability to establish a temporal sequence among stroke, depression, and fracture occurrence. Future studies should incorporate multicenter and demographically diverse cohorts to further validate the intricate relationships among these variables. A prospective study design would be beneficial, as it would allow for a more accurate assessment of the temporal relationships between stroke, depression, and fracture risk (60). This approach would help in understanding the causal pathways and potential differences in outcomes across various stroke subtypes, such as ischemic and hemorrhagic strokes. Additionally, intervention studies could be recommended to evaluate the impact of targeted mental health interventions on reducing fracture risk in stroke patients. Such studies could include randomized controlled trials to assess the efficacy of depression management programs in improving bone health outcomes and reducing fall-related injuries. Moreover, animal experiments exploring the mechanisms of the brain-bone axis could provide valuable insights into the underlying physiological processes. Studies using animal models can help elucidate the bidirectional communication between the brain and bone, involving factors such as neuroendocrine signals and extracellular vesicles. Such research could advance our understanding of neuroendocrine signals and extracellular vesicles, ultimately contributing to the development of therapeutic strategies for treating both neurological and musculoskeletal disorders. By exploring these mechanisms, researchers may pave the way for developing novel therapeutic strategies that target both neurological and musculoskeletal disorders.

## 5 Conclusion

This study indicates a positive association between stroke and fractures, highlighting depression as a substantial mediating factor. It suggests that clinicians should consider both conditions concurrently in their assessments.

## Data Availability

Publicly available datasets were analyzed in this study. This data can be found at: https://www.cdc.gov/nchs/nhanes/NHANES.
